# Contributions from Patients

**Published:** 1920-04-03

**Authors:** Alexander Pym

**Affiliations:** Secretary. The Royal Waterloo Hospital for Children and Women.


*To the Editor of* The Hospital.

### CONTRIBUTIONS FROM PATIENTS.

*To the Editor of* The Hospital.

Sir,—I am to inform you that with reference to the recent decision of the London Hospital to levy a weekly charge of 10s. on those patients who are in a position to pay such a sum, the authorities of this hospital do not propose at present to make any change in their policy. They are (and have been) accustomed to ask for voluntary contributions from suitable patients, but no steps are taken to enforce such a request. It is believed that the financial crisis is temporary, and that the whole spirit of the voluntary system is too precious to run any risk of endangering it even by the otherwise justifiable measure of adopting fixed charges.—Yours faithfully,

Alexander Pym, Secretary.

The Royal Waterloo Hospital for Children and Women.

March 24, 1920.

